# An estimation on the mechanical stabilities of SAMs by low energy Ar^+^ cluster ion collision

**DOI:** 10.1038/s41598-021-92077-3

**Published:** 2021-06-17

**Authors:** Y. Tong, G. R. Berdiyorov, A. Sinopoli, M. E. Madjet, V. A. Esaulov, H. Hamoudi

**Affiliations:** 1 Qatar Environment and Energy Research Institute, Hamad Bin Khalifa University, Doha, Qatar; 2 Max Planck Institut für Physik Komplexer Systeme, 01187 Dresden, Germany; 3 Institut des Sciences Moléculaires D’Orsay, UMR 8214 CNRS-Université, Université Paris Sud, Université Paris Saclay, bât 520, 91405 Orsay, France

**Keywords:** Materials for devices, Nanoscale materials, Structural materials, Nanoscale materials

## Abstract

The stability of the molecular self-assembled monolayers (SAMs) is of vital importance to the performance of the molecular electronics and their integration to the future electronics devices. Here we study the effect of electron irradiation-induced cross-linking on the stability of self-assembled monolayer of aromatic 5,5′-bis(mercaptomethyl)-2,2′-bipyridine [BPD; HS-CH_2_-(C_5_H_3_N)_2_-CH_2_-SH] on Au (111) single crystal surface. As a refence, we also study the properties of SAMs of electron saturated 1-dodecanethiol [C12; CH_3_-(CH_2_)_11_-SH] molecules. The stability of the considered SAMs before and after electron-irradiation is studied using low energy Ar^+^ cluster depth profiling monitored by recording the X-ray photoelectron spectroscopy (XPS) core level spectra and the UV-photoelectron spectroscopy (UPS) in the valance band range. The results indicate a stronger mechanical stability of BPD SAMs than the C12 SAMs. The stability of BPD SAMs enhances further after electron irradiation due to intermolecular cross-linking, whereas the electron irradiation results in deterioration of C12 molecules due to the saturated nature of the molecules. The depth profiling time of the cross-linked BPD SAM is more than 4 and 8 times longer than the profiling time obtained for pristine and BPD and C12 SAMs, respectively. The UPS results are supported by density functional theory calculations, which show qualitative agreement with the experiment and enable us to interpret the features in the XPS spectra during the etching process for structural characterization. The obtained results offer helpful options to estimate the structural stability of SAMs which is a key factor for the fabrication of molecular devices.

## Introduction

The saturation in electronics devices performance is due mainly to the physical limitation of the silicon technology in the nanodomain. Molecular electronics, implementing organic molecules as building blocks, has a great potential to substitute the silicon technology at the nanoscale due to exceptional physical and chemical properties that molecular systems can provide^1,2^. In this field, the molecular self-assembly is  becoming a standard tool in nanofabrication, which enables one to create interface structures with enhanced functionalities. Self-assembled monolayers (SAMs) of thiol-end molecules on noble metal surfaces are promising hybrid systems for practical applications in electronics due to high stability and outstanding electronic and transport properties. Such systems are also suitable for large scale production due to easy preparation, materials availability, and large surface coverage at low cost.

To understand the properties of the molecular systems and further improve their performance, extensive research has been conducted addressing problems ranging from the molecular architecture to the organic/inorganic hybrid interface formation. A particular focus has been given to the effects of the thickness of the SAMs, by using different types of head groups/binding groups and different molecular backbone from alkyl chain to π-conjugated aromatic structures. Thiol-gold interfaces, through S–Au covalent bonding, are of fundamental importance in SAM creation and therefore, their properties are studied using different measurement techniques, such as X-ray photoelectron spectroscopy (XPS) and scanning tunneling microscopy (STM). It has been assumed that such chemical thiol-gold bond is formed by breaking S–H bonds near the interface^3,4^. Depending on the molecular backbone, a different anchor angle between the sulfur and gold atom is realized, which further determines the orientation of the SAM arrays. X-ray photoemission measurements also reveal a binding energy shift with different bonding positions of thiol on Au (111) single crystal surface^5^. Studies on the effects of the substrate morphology on the adsorbed molecules have been conducted in terms of, molecular size, charge exchanging, electric dipolar modification, working function variation and electronic level alignment to the Fermi edge. For example, tuning the work function of a metallic substrate with a molecular electric dipole involves attaching a polar head group to the SAMs, varying the dipole of the binding group at the interface, or inserting a polar group into the molecular backbone^6,7^.

Density functional theory (DFT) is known to be an effective predictive tool in studying the formation process and properties of SAMs on metallic surfaces, and interpreting the results of XPS measurements^8,9^. For example, Taucher et al*.* used DFT in describing chemical *vs.* electrostatic shifts in XPS spectra of organic SAMs^10^. In another study, Cabarcos et al*.* investigated the influence of embedded dipole layers on the electronic properties of alkanethiolate SAMs using different experimental techniques, such as infrared reflection absorption spectroscopy (IRS), high-resolution XPS, ultraviolet photoelectron spectroscopy (UPS), atomic force microscopy (AFM), and Kelvin probe (KP) AFM^11^. The experiments were complemented with DFT-based electronic band-structure calculations to get a fundamental insight into the processes taking place in the considered systems. First principles calculations have also been successfully implemented to interpret the experimental results in studying the effect of embedded dipole moments of molecules, for interface engineering through self-assembly^12^. Recently, Berdiyorov and Hamoudi used first-principles calculations to study the effects of molecular backbone and anchoring groups on the transport properties of aromatic molecules sandwiched between metallic electrodes^13,14^.

In this work, we present a detailed study of structural and electronic properties of SAMs of aliphatic 1-dodecanethiol (C12) and aromatic 5,5′-bis(mercaptomethyl)-2,2′-bipyridine (BPD) molecules on Au (111) substrate. Well-ordered molecular arrays are obtained as revealed from the S2p core-level XPS spectra. To investigate the interface properties in detail, we apply a low energy cluster-type depth profiling method, and the system evolution is monitored by XPS on both core level of C1s and S2p, and by UPS in the valence band range. Such measurements enable us to test the stability of the molecular structures by peeling-off the molecular fragment gradually from the top of the SAMs towards the interface using atomic collision techniques. We also study the effect of electron beam irradiation on the resistance of the molecular SAMs to the etching. The stability of the aromatic molecules increases by more than 4 times due to electron irradiation-induced cross-linking of the aromatic BPD molecule. On the contrary, the stability of the C12 molecules decreases after electron irradiation. We further conduct DFT calculations to describe the phenomenon of the binding energy shift during the etching process.

## Results

### XPS measurements

**Figure 1:**
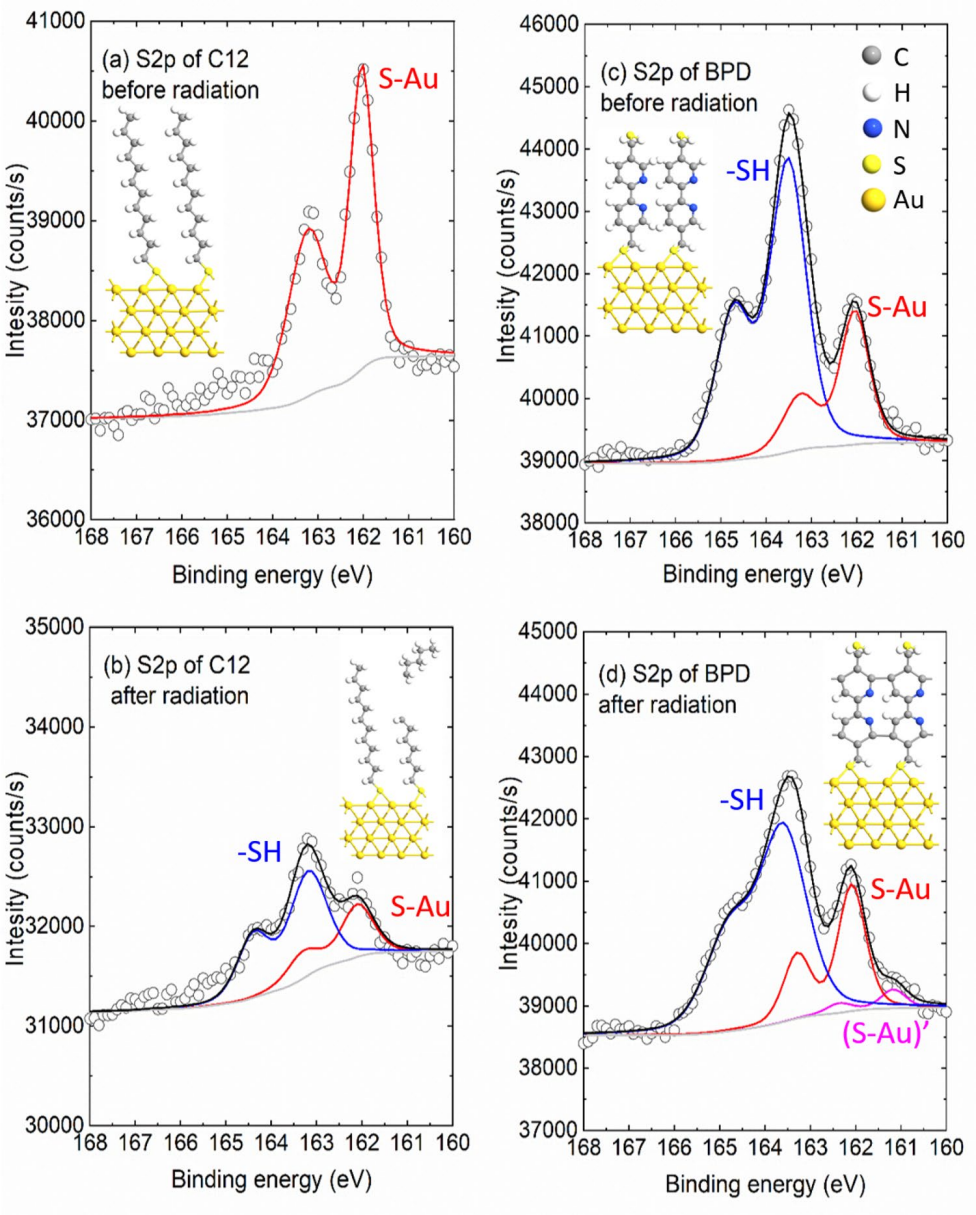
The S2p core level spectra of C12 (**a,** **b**) and BPD (**c**,**d**) SAMs before (**a**,**c**) and after (**b**,**d**) the electron beam irradiation. The intensities are normalized to the corresponding Au4f signal. Spectra are fitted with a Voigt profile after a Shirley background subtraction. The three components are utilized here: the free S–H at around 163.5 eV (blue), the S–Au bonding at around 162.0 eV (red) and another (S–Au)’ bonding position at around 161.0 eV (purple). Insets in (**a**) and (**c**) show optimized structures of C12 (**a**) and BPD (**c**) molecules on gold surface. Insets in (**b**) and (**d**) illustrates possible structural changes of C12 (**b**) and BPD (**d**) SAMs after electron irradiation.

SAMs of both C12 and BPD molecules were characterized by XPS before and after the electron irradiation through the S2p and the C1s core level spectra (see Fig. 1; Fig. S1) with a particular focus on the deconvolution of the S2p and C1s signals. XPS signals of the non-radiated C12 SAM show well defined splitting of S2p spectrum with the main maximum located at 162.0 eV (Fig. 1a), which corresponds to the thiol sulfur in the S–Au bonding cascade. No clear oxidation component (located around 168.0 eV) is observed, indicating a good structural integrity of C12 molecules. This is further confirmed by C1s spectrum, which indicates only C–C structure and almost no C=C/C–O related peaks expected at 286.5 eV (see Fig. S2a). These findings indicate that all the constituents of the C12 are attached to the Au electrode in a uniform manner. S2p core level signal of non-radiated BPD SAM is significantly different from the signal obtained for C12 SAM (compare Figs. 1a and 1c). As depicted in Fig. 1c, the amplitude of the peak at 163.5 eV becomes larger than the one at 161.9 eV. Notice that we have rinsed very well our samples after the SAM formation to avoid any physiosorbed molecules at the interface. Different papers using same system show similar results as what we obtain in the present experiment^15^. Since the BPD attached to the Au surface is oriented with a stand-up structure, the signal from the S–Au at the interface is attenuated by the molecular backbone. This is not the case for the –SH signal as it locates on top of the monolayer and photoelectrons reach the vacuum without any attenuation. This explains the relatively higher –SH signal than the S–Au despite the equal number of sulfur atoms in the BPD structure. Due to the different nature of the molecules, the C1s spectrum of the BPD molecules is also different from the one obtained for C12 molecules. The component at 284.8 eV arises from the C–C moieties, and C=C in the ring while a strong shoulder at higher binding energy of 285.6 eV was attributed to C–N signal^16,17^ (see Fig. S1).

We then test the film resistance to the electron irradiation. The electron beam was set 1000 eV in energy with an exposure time of 30 min on top of the two SAMs. A direct comparison of the C1s and S2 spectra before and after electron irradiation is shown in Fig. S2 in the supporting information. It is seen from this figure that C1s signal of the C12 SAM exhibits a great loss in intensity (almost 50%, see Fig. S2a) and the S2p signal gets distorted both in intensity and the profile. On the contrary, in the case of the BPD SAM the effect of the electron irradiation on both C1s and S2p spectra is much less pronounced; the reduction in the signal amplitude is less than 10% (see Fig. S2c,d). We specifically clarify such evolution by looking into the S2p deconvolution. As shown in Fig. 1b, the irradiation induces a significant signal loss of the S2p signal of the C12 SAM together with appearance of a new component in the spectrum at 163.1 eV which is assigned to a –SH. This clearly indicates that the electron irradiation cause S–Au bond breaking at the interface, leaving the thiol-like fragment unbounded to the substrate and appearing as the signal at 163.1 eV. On the other hand, BPD is quite resistant to the electron irradiation as no clear variations are obtained in the S2p spectrum (see Fig. 1d), although a small new component in the spectrum at 161.1 eV appears. This small energy component is related to thiolate-Au bond but at a second adsorption position^5^. Briefly, molecules form dense SAM due to non-bonding intermolecular interactions to provide minimum space packing between the molecules. However, electron irradiation leads to intermolecular cross-linking, i.e., it generates bonding between the molecules due to the electron attachment/detachment phenomenon. This will reorganize completely the topology of the molecule at the interface, leading to the decrease of the intermolecular spacing and create new adsorption sites for the thiol on Au at the second adsorption position. The relative intensity of the 163.5 eV component is slightly reduced when referenced to the 161.9 eV component.

### Depth profiling analysis

**Figure 2:**
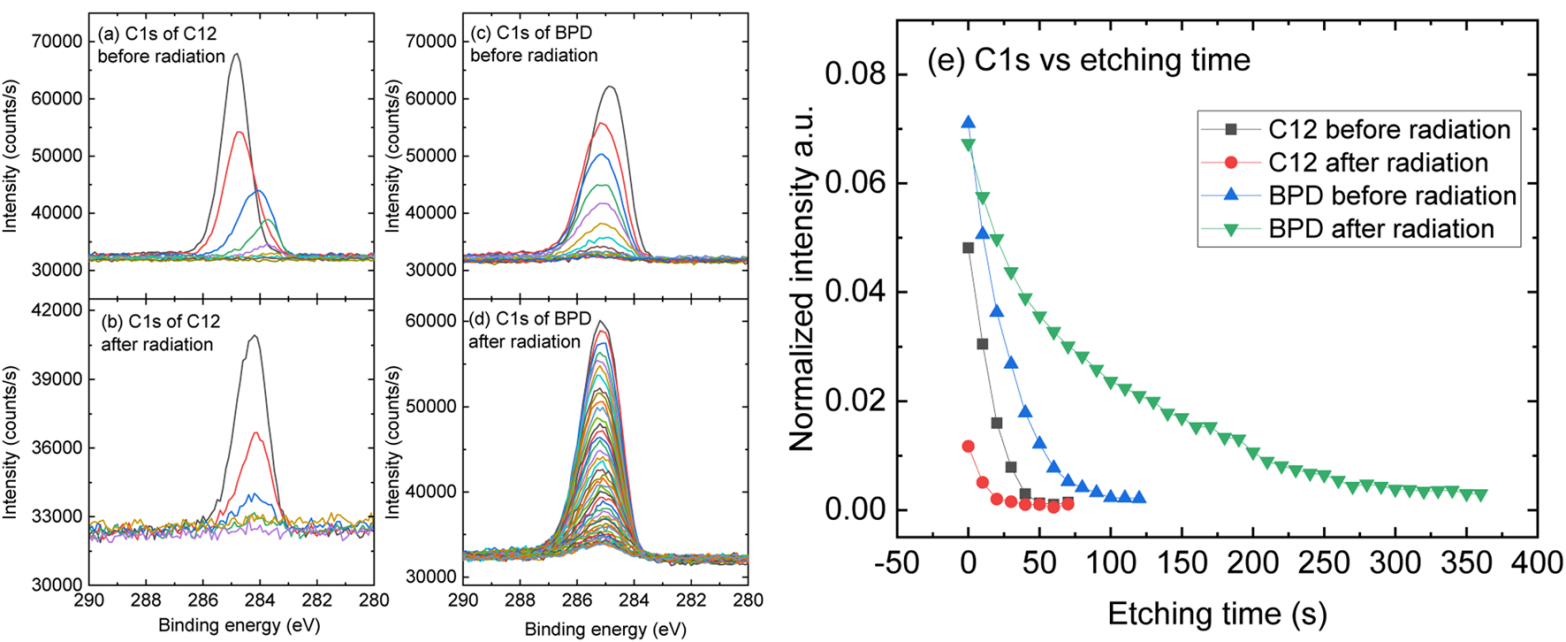
(**a**–**d**) Evolution of C1s core level spectra as function of etching time for C12 (**a**,**b**) and BPD (**c**,**d**) SAMs before (**a**,**c**) and after (**b**,**d**) the electron irradiation. The C1s signals are attenuated with increasing number of etching levels, which are presented from top to bottom (etching time ranging from 0 to 60 s for C12 and 0–160 s (0–350 s after the irradiation) for BPD). (**e**) Normalized intensities (normalized with the substrate Au4f signal at the respective etching level) of C1s spectra as a function of the etching time.

We further test the stability of the considered SAM structures by performing depth profiling analysis before and after the electron irradiation etching by low energy Ar^+^ cluster ions incident at 45° to the surface and by recording both the UPS and the C1s/S2p core level spectra. Figure 2a–d shows the C1s core level spectra of C12 (a, b) and BPD (c, d) during the etching process before and after electron irradiation. The relatively low energy Ar^+^ clusters enabled the removal of molecular fragments from the top of the molecules gradually, leading to the intensity of the C1s attenuating as a function of the etching time. Such attenuation turned out to be more pronounced in the case of C12 molecule than BPD ﻿by compar﻿ing Fig. 2a,b. Another interesting phenomenon we observed is the clear binding energy shift with etching. In the case of C12 SAM, the maximum of the C1s binding energy, initially located at 284.8 eV, shifted progressively to lower binding energy up to 283.8 eV at the last stage (see Fig. 2a). Thus, a red shift of 1 eV is obtained for this molecular SAM. On the contrary, in the case of the BPD SAM (Fig. 2c), the maximum of the C1s signal first shifts slightly to higher energy (a slight blue shift) in the first few etching levels and then starts shifting to lower energies (redshift). The etching behaviors after the electron irradiation for C12 and BPD SAMs are shown in Fig. 2b,d, respectively. In the C12 case, the irradiation-induced structural deterioration can be deduced as it costs only 3 etching periods (totally 30 s) to completely remove the C1s signal (see Fig. 2b), compared to 60 s to achieve the same effect on the as-assembled C12 SAMs (Fig. 2a). On the contrary, the BPD SAM after the irradiation is quite resistant to the Ar^+^ beam with the same energy: while it needs 120 s of etching on the pristine BPD SAM (Fig. 2c), a total of 360 s is applied until the BPD SAM signal is mostly removed after the irradiation (Fig. 2d).

Figure 2e summarizes our findings regarding the effect of electron-irradiation on the stability of the considered SAM structures, where we plot the intensities of the C1s signal normalized to the Au4f signal at each etching level as a function of the etching time. From the comparison, one can clearly observe the intensity loss and structure deterioration for C12 SAM upon the irradiation. For example, 50 s of etching time is needed for complete removal of the as-assembled C12 molecules from the substrate (black squares in Fig. 2e), whereas it takes 25 s to remove the molecules after electron irradiation (red circles in Fig. 2e). In addition, the C1s signal intensities are always smaller in the latter case. The C1s signal of as-assembled BPD molecules are observed up to 100 s of etching time (blue triangles in Fig. 2e), which is twice longer than the one obtained for the C12 molecules. Interestingly, the etching time required for the removal of the BPD SAM increases dramatically after electron irradiation: C1s signal is observed even after 350 s of etching (green triangles in Fig. 2e). Such an enhanced stability is related to intermolecular cross-linking of the BPD molecules during the electron irradiation^18^.

**Figure 3:**
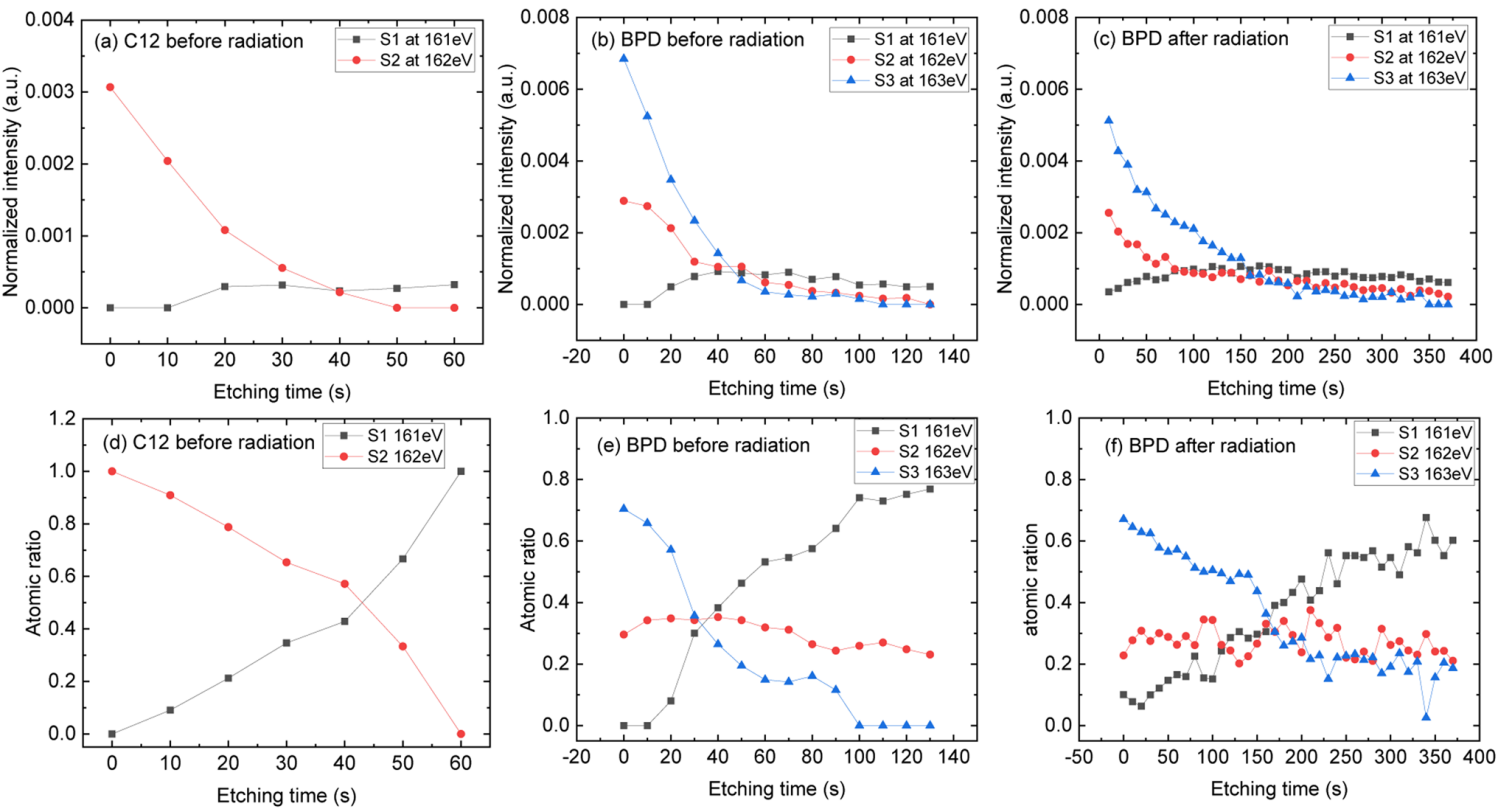
(**a**–**c**) Intensities (normalized to the Au4f signal) of the components of S2p spectra of pristine C12 SAM (**a**), pristine BPD SAM (**b**) and irradiated BPD SAM (**c**) as a function of etching time. The same results are presented in (**d**–**f**) normalizing the signal intensities to the total signal intensity to see the percentage contribution of different components. S1, S2 and S3 corresponds to the S2p3/2 at 161.1 eV (component S1), 162.0 eV (component S2) and at 163.5 eV (component S3), respectively.

The evolution of S2p spectra as function of etching time is shown in Fig. S3 in the supporting information, which also shows the similar phenomenon discussed above. However, in the S2p case we witness a continuous signal fading with etching level. Therefore, we fit the S2p signal at all etching intervals (as explained in Fig. 1) and present in Fig. 3a–c the intensities of the components with the maximum of the distributions at 163.5 eV (component S3), 162.0 eV (component S2) and at 161.1 eV (component S1). The intensities are normalized to the Au4f signal. These components are assigned to the –SH headgroup, and the S–Au bond at two different positions, respectively. For better representation, we also plot the percentage composition of the signals by normalizing the data to total intensity (i.e., atomic ratio). The results are shown in Fig. 3d–f. In the case of C12 SAM, the S2 component contributes most to the spectrum at smaller etching times (red circles in Fig. 3a,d). With increasing etching time, the contribution of the S1 signal becomes more pronounced (black squares in Fig. 3a,d). Similar results are obtained for the C12 SAM after the electron irradiation. However, the amplitudes of the signals become less pronounced with etching time (see Fig. S3). In the case of pristine BPD SAM (Fig. 3b,e), the largest signal at small etching times correspond to S3 component of the spectrum (blue triangles) followed by the S2 component (red circles). With increasing etching time, the intensities of both S3 and S2 components decreases, whereas the intensity of the S1 component increases (black squares). The intensities of all components vary weakly with etching time at later etching times. Similar dependence of the intensities of the S2p signal components is obtained after the electron irradiation (see Fig. 3c,f). The difference is that the signals are observed at larger etching times. Compared with the Fig. 3b,c, the irradiation causes mainly the loss of the S3 component, which is associated with the desorption of alkane chain fragment induced by the irradiation. Note also that the etching mainly affects the S3 and S2 component but leaves the S1 residual almost constant in the later etching intervals.

**Figure 4:**
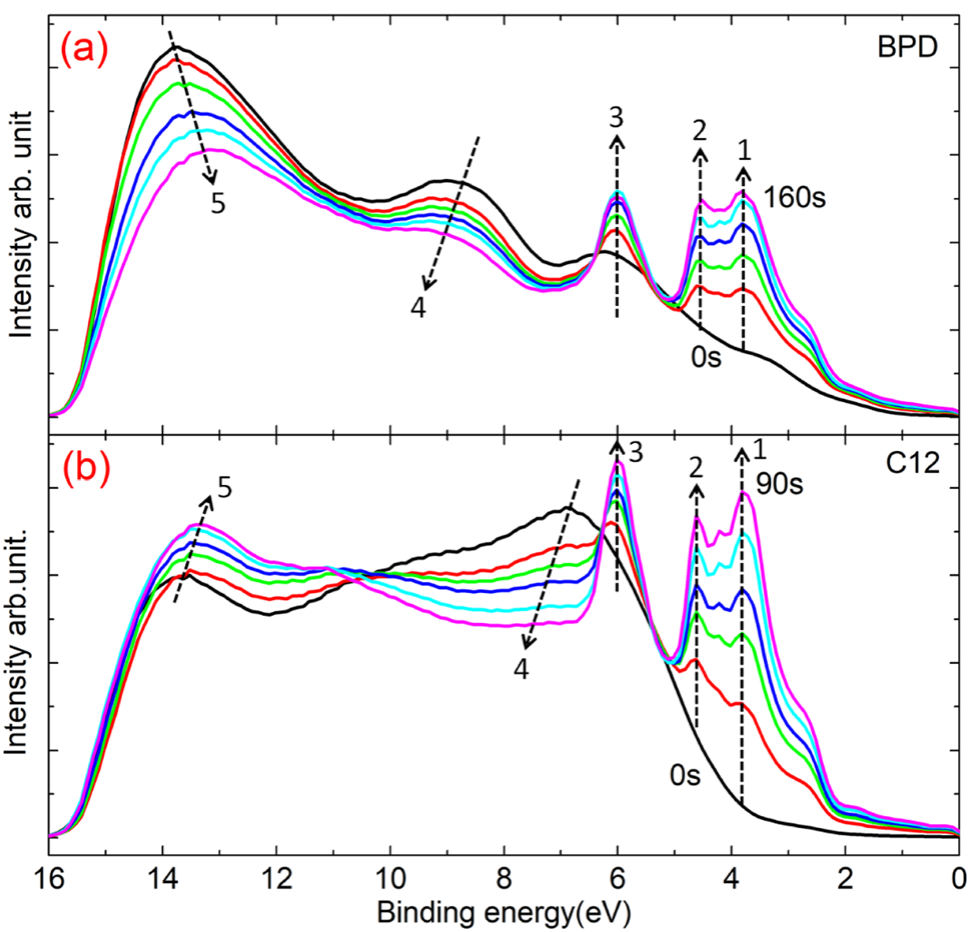
UPS spectra of as-assembled BPD (**a**) and C12 (**b**) SAMs for different etching times ranging from 0 to 90 s for C12 and 0 to 160 s for BPD SAMs. The etching times are 0 s, 10 s, 20 s, 30 s, 50 s, 90 s for C12 and 0 s, 20 s, 40 s, 70 s, 100 s, 160 s for BPD. The complete spectra are given in Fig. S5. Vertical dashed lines indicate the change in the intensity and the energy shift (if any) with etching time.

We have also conducted UPS measurements during the etching process for the considered systems. Figure 4 shows the UPS spectra of as-assembled BPD (a) and C12 (b) SAMs at different etching times (see Fig. S4 for the UPS spectra of both samples after electron irradiation). It is clear from this figure that the effect of ion beam etching on the features of the UPS spectra depends both on the type of the molecules and the binding energy values. At low energies (< 7 eV), regardless of the type of molecules, the amplitude of the UPS spectra increases with etching time, showing several additional maxima in the UPS spectra. Interestingly, the locations of those UPS maxima do not change with etching (see vertical arrows 1–3 in Fig. 4). We attribute these changes in the UPS signal to the strong contribution of Au-substrate, which is initially screened by the molecular layer. This also explains the fact that the position of the UPS maxima, at these low energies, does not change with etching. The reduction in the “screening” effect is more pronounced in the C12 system, indicating that C12 molecules get more damaged during the ion bombardment. The situation changes considerably at higher (> 7 eV) binding energies, where, for both samples, the amplitude of the UPS maxima decreases, while their position shift to higher energies, with increasing the etching time (arrows 4 in Fig. 4). With further increase of the binding energy, a UPS maximum is obtained around 13.5 eV for both samples.

**Figure 5:**
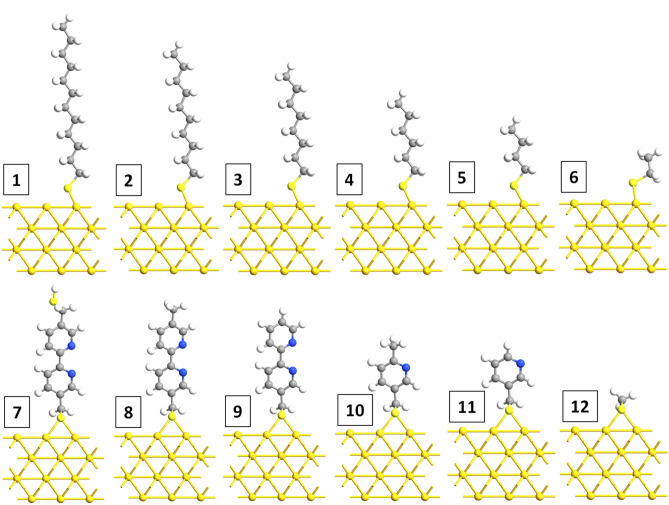
Model systems: optimized structures of C12 (panel 1) and its derivatives (panels 2–6) and BPD (panel 7) and its derivatives (panels 8–12).

To understand the features on the UPS spectra during the etching process, we have conducted DFT simulations for C12 and BPD molecules and their possible derivatives. Figure 5 shows our model systems for C12 (panels 1–6) and BPD (panels 7–12) derivatives. The selected structures were optimized using periodic boundary conditions along the lateral directions, and large vacuum regions perpendicular to the gold (111) substrate. The coordinates of the atoms in the last layer of the gold substrate were kept fixed during the optimizations to represent the bulk structure. All molecules are attached to the substrate through S–Au covalent bonding. For both types of molecules S atoms are bound to 2 neighboring gold atoms (i.e., bridge geometry bonding).

**Figure 6:**
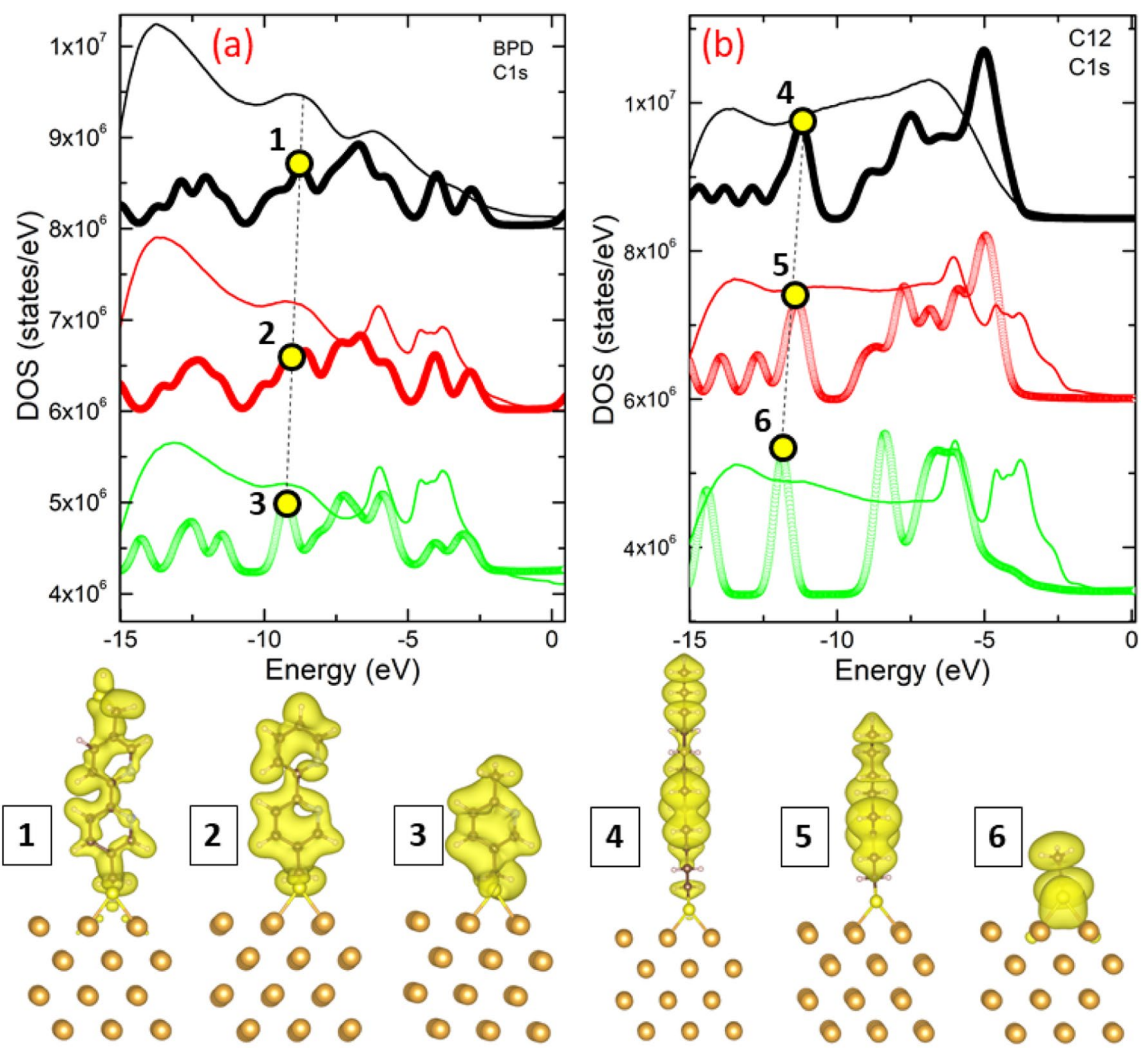
Calculated DOS (thick curves) of (**a**) BPD derivatives (panels 1–3) and (**b**) C12 derivatives (panels 4–6) as a function of energy normalized to Fermi energy. Thin curves show the experimental UPS spectra at different etching times. Panels 1–6 show the isosurface plots of electron eigenstates at electron energies indicated on the DOS curves. Panels 1–3 in this figure are the model systems presented by panels 7, 9 and 10 in Fig. 5. Panels 4–6 in this figure correspond to panels 1, 3 and 6 in Fig. 5, but viewed from different angle.

We attempt to use the simulated models to explain the shift observed in the UPS spectra of the considered systems indicated by dashed arrows 4 in Fig. 4. Figures 6a,b show the calculated density of states (DOS) for 3 BPD-based structures (thick curves in Fig. 6a and panels 1–3) and 3 C12-based structures (thick curves in Fig. 6b and panels 4–6). As comparison, we also present the experimentally obtained UPS signals (thin curves in Fig. 6). Dashed black line in Fig. 6a highlights the blue shift of the experimental UPS spectra at different etching levels. The calculated DOS (solid thick lines in Fig. 6a) shows a similar pattern as in the experiment. For example, the calculated DOS of the system also shows a blue shift in this range of the energy. To see the contribution of the electronic states to the DOS of the system and to understand the nature of the peaks on the UPS spectra, we have calculated electronic eigenstates for different energy values. Panels 1–3 in Fig. 6 show the electron density of the states corresponding to the peaks on the DOS curves indicated by symbols 1–3 in Fig. 6a. As expected, only the electron density of the adsorbed molecules contributes to the DOS of the system for the considered energy values. Therefore, the peaks shift to higher energies because the binding energy of smaller fragments becomes higher. At low energies, the gold atoms strongly contribute to the DOS of the system. The positions of the maxima on the DOS curves does not change with decreasing the molecular length in this range of the energy which is in good agreement with the experimental data. Similar dependence of the DOS on the length of the molecule is also obtained in the case of C12 molecules (see Fig. 6b and panels 4–6). The only difference is that the contribution of the substrate atoms to the DOS of the system is stronger as compared to BPD system. This is also in good agreement with the experiment. Notice that in our model systems we did not consider the effect of cross-linking on the electronic properties of BPD molecules. This is due to complexity of modelling the process of molecular decomposition after the intermolecular cross-linking. Notice also that we did not consider the case when the molecular fragments are reattached to the substrate (or to the molecule itself), which can be considered as an ideal case of molecular fragmentation.

## Discussion

The response of molecular SAMs to the ionizing radiation (e.g., electron irradiation and X-ray exposure) is important characteristics for molecular systems as it determines the performance of the molecular devices working under similar environment. The modification of the SAMs induced by the irradiation has been reported, including decomposition of the SAM’s entities^19–21^, breaking of the molecular fragment^22,23^ or headgroup to substrate attaching^24–26^, reorientation/disordering^27^, and deformation^28^, e.g., cross-linking^29–31^. It has been proven that such effect greatly depends on the molecular architecture in both the structural and electronic properties. In the present case, C12 is an alkane like molecule with all the electron localized throughout the C–C bond. The intermolecular interaction is dominated by the weak Van de Waals force, leaving the entire film quite fragile to the Ar^+^ ion beam. Moreover, its saturation in electron density makes it quite sensitive to the electron bombardment. Consequently, it is expected to induce structural deterioration, desorption of the alkane fragment and eventually the breaking of the S–Au at the interface^30^*.* This phenomenon is indeed observed in our experiments during the etching process as shown in Fig. 2e. This is also evidenced from Fig. S2 where the carbon content greatly attenuates in the C1s spectrum and the new signal appears corresponding to the sulfur component around 163.1 eV in the S2p spectrum upon electron irradiation. On the contrary, aromatic structure containing the π electrons gains a better structural stability towards Ar^+^ bombardment. The π–π interaction between molecules makes the film steadier as compared to the alkane C12. The BPD molecules behave quite resistant to the electron irradiation. This is due to the electron delocalization in the molecular plane that allows the redistribution of the coming electrons and before the molecules become electron saturated. Thus, the whole molecule acts as molecular conductor that enable the transportation the extra electron to the Au substrate, only the weak bonds can be cut such as the C–H bond, which is the origin of the cross linking between the molecules. This explains the high resistance of the BPD SAMs towards the electron irradiation. More importantly, we also observe a mechanical stability reinforcement of the BPD SAMs through electron irradiation. The irradiated BPD appears quite resistant to the Ar^+^ bombardment under the same energy condition. Different from the fragment desorption of the C12, the electron beam induces the replacement of π to π intermolecular interaction by a more stable C–C bond through the cyclic carbons after the C–H bonds scission^29^. Such intermolecular cross-linking makes the film behave quite similar to a 2D carbon structures^32^ and can explain the much longer depth profiling process.

As we have mentioned above, at low energies (0–7 eV) the UPS features from the underlying alkyl chain of the C12 molecules and aromatic parts of the BPD molecules are gradually replaced by the photoionization features of the gold substrate as the length of the molecules decreases. This effect of the substrate is more pronounced in the case of C12 SAM. The position of the maxima of the UPS spectra does not change with etching in this range of the energy. In the region between 12.0 and 17.0 eV we see decreasing in the intensity in the background of scattered secondary electrons that rise initially from photoemission in the alkanethiol layer, as well as in the gold near-surface region. In both cases, we observed a clear shift to low binding energy in the cutoff energy region between 12.0 eV and 17.0 eV as the molecular length decrease.

**Figure 7:**
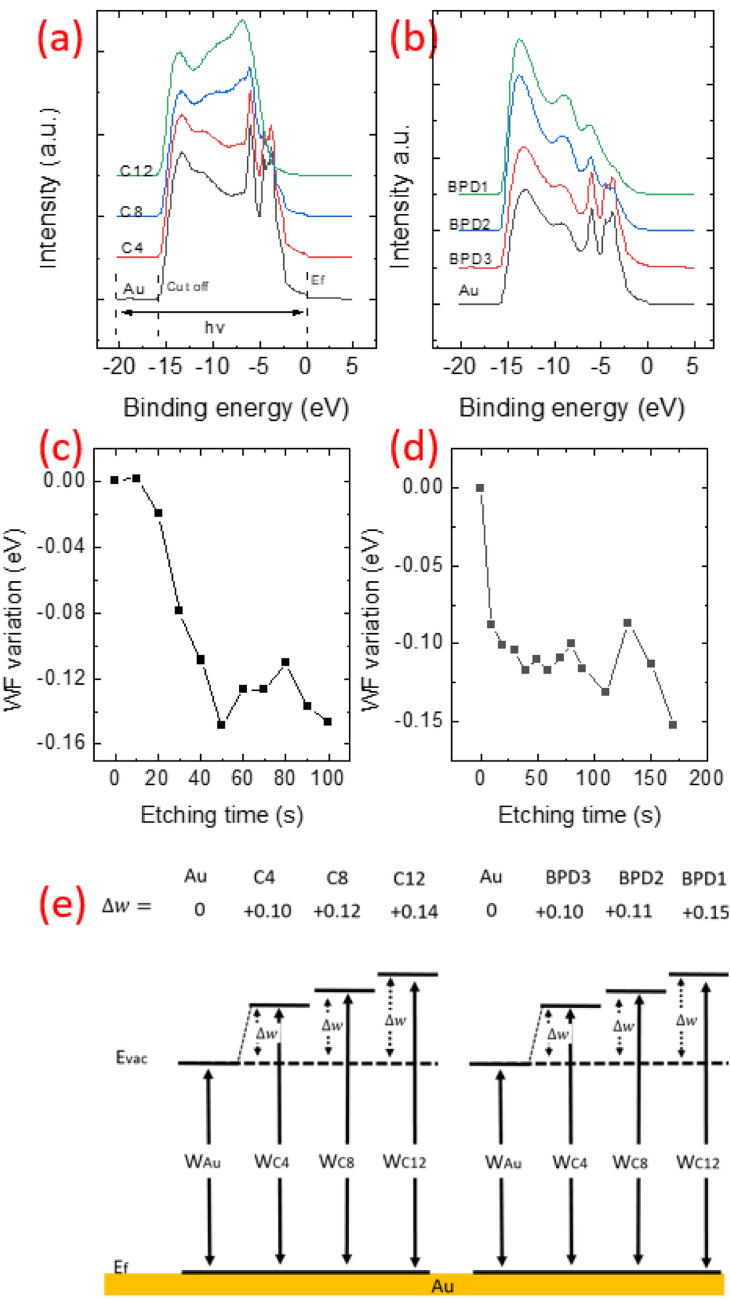
(**a,** **b**) UPS signals of C12 (**a**) and BPD (**b**) SAMs. (c, d) The Work function (WF) variations of the C12 (**c**) and BPD (**d**) as a function of the etching time. C2, C4 and C8 represent steps at different molecular lengths as described in Fig. 5, the same is for BPD1, BPD2 and BPD3 (with BPD1 the integrated BPD layer at 0 s etching level; BPD2 an intermediated stage of 30 s, BPD3 BPD remaining fragment at final etching level of 160 s). The cut-off and Fermi level were taken from the UPS spectra for work function calculations. (**e**) Schematic representation of work function variations for the considered systems.

To explain this shift, we first calculate the work function (Φ) by looking at the cutoff and Fermi level in the UPS spectra during the etching process. We picked out the simulated spectra of C12, C8 and C4 for the C12 (indicated in Fig. 7a) and BPD at similar etching stages. In Fig. 7b we show the initial spectra (BPD1 at 0 etching time) and BPD2 and BPD3 after 30 and 160 s of etching. We also show the bare Au (black line) as a comparison. Knowing the source energy *hv*, the cutoff and fermi level E_f_ was measured to obtain the work function ﻿Φ with the following equation:

$$\Phi = h\upsilon - \left(Cutoff -E_{f}\right)$$

With the value before etching as a base point, the ﻿Φ variation is plotted in Fig. 7c,d for the considered SAM structures. In both cases, an exponential drop of the ﻿Φ occurs at the initial etching stage due to the reduction of the molecular length during the etching process. With further increasing the etching time, a saturation of the work function is obtained (with some fluctuations) due to the dominant contribution of the Au states. We correlate the ﻿Φ variation to the possible molecular structure in Fig. 7e. The ﻿Φ variation starts from 0.1 eV for short C4 structure and reaches a maximum variation of 0.15 eV for the C12 layer. Similar values are also obtained for the BPD case where a maximum of 0.15 eV is obtained for the BPD1 case. However, the relatively small variation cannot fully explain the large energy shift of the XPS C1s during the etching process.

The difference in the screening effect of photoemitted holes by the substrate has been reported in the literature. An early work by Zharnikov et al. compared the effects coming from the different lengths of the molecules and from the different substrates^9^. The binding energy position of C1s spectrum shifts slightly upwards with increasing the length of the molecular backbone from C12 to C20. This was attributed to the reduced screening of the core holes from substrate with increasing the molecular length, hence the substrate –molecule separation^33^. A similar phenomenon was also observed in other thiolate carbon–metal systems^29^. Experimentally, such enhanced hole screening effect for shorter molecular film resulted in a shift of around 1 eV^34^. The value of the binding energy shift in our experiments (see Fig. 2) is in good agreement with these findings.

In conclusion, the structural stability of alkane thiol (C12) and an aromatic dithiol (BPD) molecule self-assembled on gold (111) surface is examined using low energy Ar^+^ cluster bombardment. This method enabled us to continuously peel off the molecular fragment from the SAMs-ambient interface to the thiol-Au interface. The etching processes is monitored by recording both the C1s and S2p core level XPS spectra and UPS spectra in the valance band. In the C12/Au system, the long chain like structure for C12 exhibits a poor resistance to the mechanical peeling off by the ion beam. After exposure to the electron beam, the decomposition of the alkane chain with subsequent desorption of the organic fragment and the cleavage of the thiol-Au bond are observed at earlier etching times. Both C1s and S2p signal significantly attenuates and alkylsulfide species newly appear in the S2p profile. In contrast, in the BPD/Au system, the irradiation-induced process involves the intermolecular crosslink via the cyclic C–C bond, which results in an enhancement of the mechanical stability of the system. This leads to much longer depth profiling with Ar^+^ ion collision under same experimental conditions. We assign this high resistance towards the Ar^+^ collision to the contribution from the π–π intermolecular interactions via the aromatic rings. Compared to the weak Van de Waals force, π stack like structure can significantly maintain the structural integrity of the SAMs during mechanical damage. Another advantage of the BPD structure lies in its high stability  to the electron beam irradiation. Such resistance is attributed to its electron delocalization property. The BPD acts like a molecular conductor during electron irradiation that transports the external electrons to the Au electrode. Meanwhile, the C–H scission enables also the bonding of the cyclic carbons between the neighboring molecules, forming the 2D like structure that significantly enhance the structural stability. This leads to a pronounced extension of the depth profiling process.

## Methods

### Sample preparation

All the chemicals and solvents were purchased from Sigma Aldrich and used for sample preparation without further purification. Pure ethanol and hexane were degassed under nitrogen gas flow for 30 min prior to their use. Gold (111) on mica substrates were purchase from PHASIS Switzerland, rinsed with absolute ethanol and dried under nitrogen gas flow prior to use. SAM structures are created following the procedure described in Ref.^35^ as follows. C12@Au SAM was prepared using 1 mM solution of C12 in absolute ethanol. The solution was degassed for 30 min before immerging the gold substrate. The system was kept for 4 h under reduced light condition and nitrogen blanket. After that, the gold substrate was washed three times with absolute ethanol and dried with nitrogen gas. BPD@Au SAM was prepared by using 1 mM solution of BPD in hot hexane (60 °C). The solution was maintained at 60 °C and degassed for 30 min before immerging the gold substrate. The system was kept for 1 h, under reduced light condition and nitrogen blanket. After that, the gold substrate was washed once with hexane and three times with absolute ethanol, then dried with nitrogen gas. Surface analysis of the studied SAMs were performed immediately after the sample preparation, to avoid oxidation of the adsorbed molecules.

### Photoemission

The photoemission measurements were performed on the standard Thermo Fisher ESCALAB 250XI type XPS platform. A monochromatic Al Kα Anode X-ray beam of 1486.6 eV was used with an energy resolution of 0.5 eV. The XPS spectra were obtained with a normal emission and a beam incident angle of 45° to the surface normal. All the energy values were calibrated with respect to the Au4f located at 84.0 eV. UPS measurements were conducted with a He discharge lamp with a beam energy of 21.2 eV. A perpendicular angle was applied for data acquisition and bias was given for the cutoff measurement. UPS values were calibrated to the Au Fermi level.

### Depth profiling and electron irradiation

The depth profiling was performed with a MAGCIS type ion gun. The procedure was conducted with a low energy Ar^+^ cluster that enables a gentle etching of the molecular layer at atomic level. The etching beam arrives at the surface at 45° to the surface normal. The ion beam was set at 2000 eV that evenly distributed on a total cluster group of 2000 Ar^+^ ions, with a beam current of 10 µA. The electron irradiation exposure of the sample is performed by the Reflective electron energy loss spectroscopy (REELS) mode with an electron beam of 1000 eV. The dose density is evaluated by a faraday cup and a value of ~ 70 × 10^–7^ C/(cm^2^ s) is calculated. The exposure time was 30 min.

### DFT simulations

Structural optimizations and electronic structure calculations were performed using DFT within the generalized gradient approximation of Perdew-Burke-Ernzerhof (PBE)^36^ to account for the exchange–correlation energy. The van der Waals interactions were taken into account using Grimme’s DFT-D3 empirical dispersion correction to the PBE^37^. The projected-augmented wave (PAW) formalism was employed to treat the core-valence electron interactions. The valence states were expanded in a plane-wave basis set with an energy cutoff of 500 eV. A k-point sampling of 6 × 6 × 1 was used for the Brillouin zone integration^38^. The calculated density of states was broadened by a Gaussian width of 0.4 eV. All calculations were performed using the Vienna Ab initio Simulation Package (VASP)^39^. In order to calculate and visualize the electron density of a given state (in the conduction or valence band), we first performed a single point calculation. The states corresponding to a given energy value were then identified. Partial charge and electronic density for selected states were calculated and converted to cube format to be plotted with the VESTA package^40^.

## Supplementary Information

Supplementary Information 1.

## Data Availability

Data is available from the authors upon request.
