# Supplementary material for: An estimation on the mechanical stabilities of SAMs by low energy Ar+ cluster ion collision
Source: Sci Rep. 2021 Jun 17;11:12772. doi: 10.1038/s41598-021-92077-3 (PMC8211834; doi:10.1038/s41598-021-92077-3)
Supplement: Supplementary file 1 — Supplementary Information 1. [file 41598_2021_92077_MOESM1_ESM.docx]

**Supplementary information**


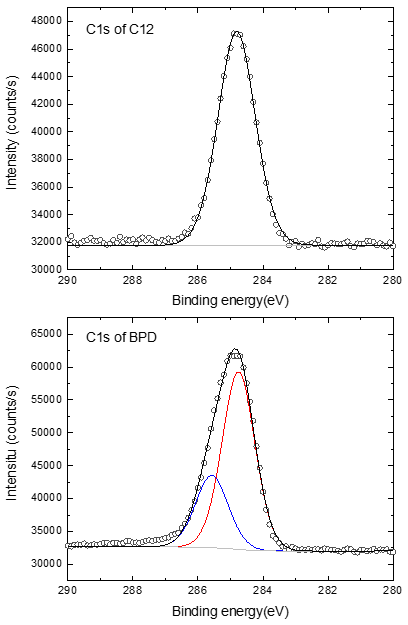


Figure S1. Fitting of the C1s for C12 and BPD. C12 gives only a C-C structure and almost no C=C/C-O related peaks above 286eV are observed. The BPD gives two components corresponding to the C-C and C-N structure, respectively.

In Figure S1 (a) C1s of C12 show good integrity with a FWHM of 1.24eV while in Figure S1 (b)the BPD case, two components is present with the FWHM of 1.17eV, relating to the C-C and C-N respectively.


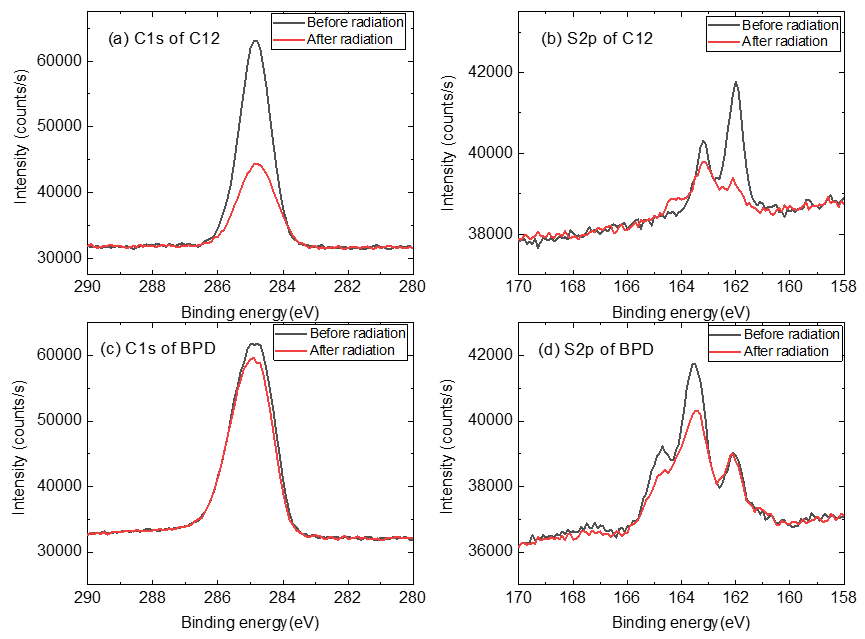


Figure S2. A direct comparison of the C1s and S2p spectra before and after the electron beam radiation. The intensities are normalized to the A4f signal.

Figure S3. Evolution of S2p during the depth profiling for the C12 (a,b) and BPD (c,d) SAMs before and after the radiation. Curves represent an etching level of 10s with the cluster-type mode. It takes 50s totally to remove the C12 layer, while 120s (curves from bottom to top) and 360s is needed for the BPD before and after the radiation, respectively. The normalized intensity vs etching time is given in (e).

Figure S4.the UPS for the C12 and BPD after radiation.

Figure S5. The UPS of C12 and BPD during the etching process.

Figure S5. The UPS of C12 and BPD during the etching process with time interval of 10 s.
